# Arthroscopic Ankle Arthrodesis for Treating Osteoarthritis in a Patient with Kashin-Beck Disease

**DOI:** 10.1155/2014/931278

**Published:** 2014-10-02

**Authors:** Kenjiro Iwasa, Noriyuki Kanzaki, Takaaki Fujishiro, Shinya Hayashi, Shingo Hashimoto, Ryosuke Kuroda, Masahiro Kurosaka

**Affiliations:** Department of Orthopaedic Surgery, Kobe University Graduate School of Medicine, 7-5-1 Kusunoki-cho, Chuo-ku, Kobe 650-0017, Japan

## Abstract

Kashin-Beck disease (KBD) is an endemic degenerative osteoarthritis. Death of cartilage and growth plate is the pathologic feature; therefore, KBD involves skeletal deformity and often results in osteoarthritis. Deficiency of selenium, high humic acid levels in water, and fungi on storage gains are considered the cause of KBD. The most frequently involved joints are ankles, knees, wrists, and elbows and symptoms are pain and limited motions of those joints. The main treatments for KBD are rehabilitation and osteotomy to correct the deformities because preventive treatment has not been established. In this report, we present a case of ankle osteoarthritis due to KBD and first describe arthroscopic ankle arthrodesis for treating osteoarthritis of KBD.

## 1. Introduction

Kashin-Beck disease (KBD) was first reported as an endemic degenerative osteoarthritis in 1919 [1], and this condition affects more than one million individuals [2] in a limited endemic area in China, Central China, from Southeastern Siberia to North China, and North Korea [3]. Death of cartilage cells in the growth plate and articular surface is the basic pathologic feature, and this can result in growth retardation and secondary osteoarthrosis [4]. Skeletal deformation begins in childhood, and the epiphyseal growth plate and articular cartilage are the most commonly affected sites [5]. The cause of KBD remains unknown [6], but deficiency of selenium [7], high humic acid levels found in drinking water [8], and fungi on storage grains are considered the causes of KBD [9]. Recently, variants in the chromosomal short tandem repeats also have been found to be associated with KBD [10]. The symptoms of KBD include joint pain, morning stiffness in the joints, and limited motion in many joints of the body [11]. The most frequently involved joints are the ankles, knees, wrists, and elbows, in which both sides are involved [12]. Because diagnosis is difficult, clinical and radiological examinations are the best for identifying KBD until recently [13]. Preventive treatment has not been established, although Zhao et al. have reported that selenium supplements could prevent and control KBD in children [14]. Therefore, physical treatments and osteotomy to correct the deformities that are found in advance cases are common treatments for KBD [12]. Ankle arthrodesis is one of the most used treatments of end-stage ankle arthritis while arthroscopic ankle arthrodesis is gaining popularity as a treatment option since its initial use by Schneider in 1983 [15].

In this report, we describe the case of a 29-year-old man who suffered from ankle pain on both sides because of KBD. Both his ankles were at end-stage osteoarthritis, and we treated him with an arthroscopic ankle arthrodesis on the right side.

## 2. Case Report

A 29-year-old man from China had been suffering from bilateral ankle pain for approximately 3 years. He did not have trauma or a relevant past history. Symptoms had disappeared eventually but had later recurred approximately 1 year ago. He visited a nearby hospital for treatment, but the doctor had difficulty diagnosing and treating his condition. The patient visited our hospital for consultation and, subsequently, he was admitted.

On physical examination, the patient's foot and gait appeared normal. He experienced pain on initial walking or walking for prolonged periods. No swelling, redness, or local heat was detected on his both ankles, but dorsiflexion of his right ankle was limited. No instability was observed in both ankles, but the pain was noted during internal rotation of his right ankle.

**Figure 1:**
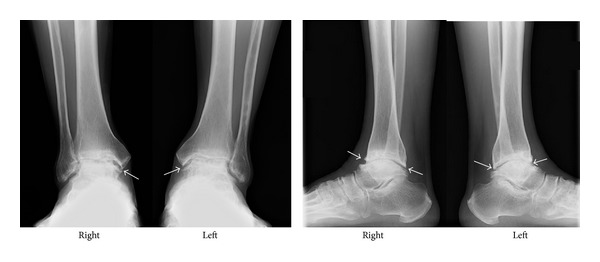
Preoperative radiographs of both the ankles. Arrows show osteophytes. Joint spaces are decreased. No pathological changes around the epiphyses plate are observed.

**Figure 2:**
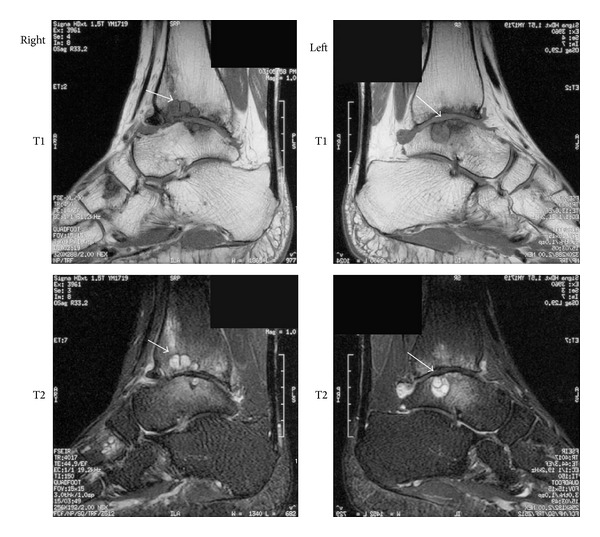
Magnetic resonance imaging (MRI) of both the ankles. Arrows show cystic lesions in both the tibia and talus. Degenerative change is also seen in right cuneiform.

Radiograph of the ankle under weight-bearing revealed that the joint spaces had decreased in weight-bearing areas of both the ankles and that osteophytes had formed mostly in the anterior site of the joints. Moreover, cystic areas were observed in these weight-bearing areas of the tibia, but no pathological changes around the epiphyses plates were observed (Figure 1). No deformities or narrow joint spaces were observed in radiographs of other joints such as fingers, wrists, knees, or hips. Magnetic resonance imaging (MRI) revealed many cystic lesions in both the tibia and talus (Figure 2). Other inflammatory diseases such as rheumatoid arthritis or infection were ruled out through blood examination results. The patient's condition was diagnosed as KBD because of the area from which he originated, X-ray and MRI findings, and the exclusion of other inflammatory diseases.

**Figure 3:**
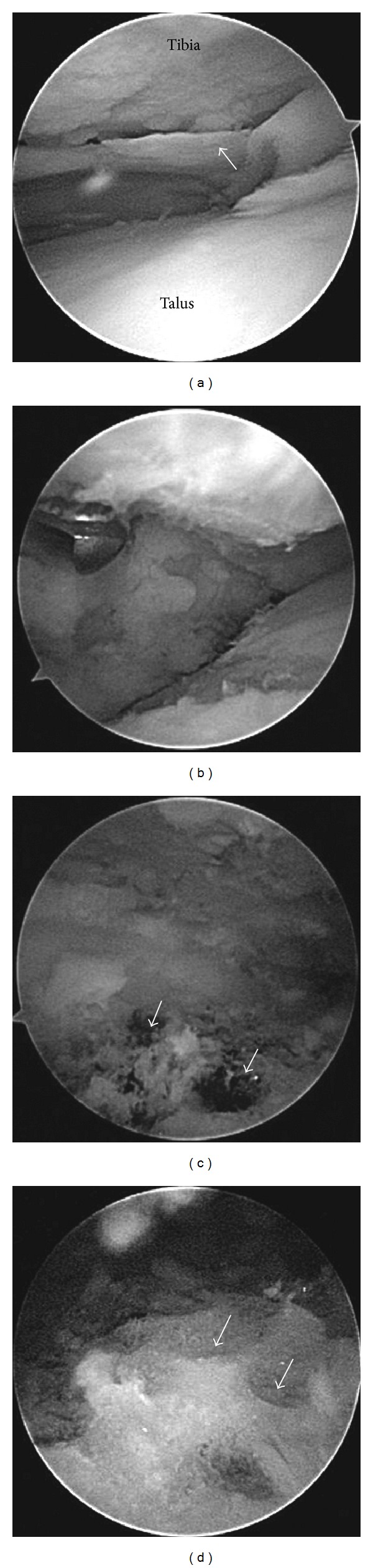
Intraoperative findings. (a) Arrow shows peeled cartilage from tibia. (b) The observed cartilage is removed using a shaver. (c) Arrows show bleeding from the dimple. (d) Arrows show numerous dimples on the talus.

**Figure 4:**
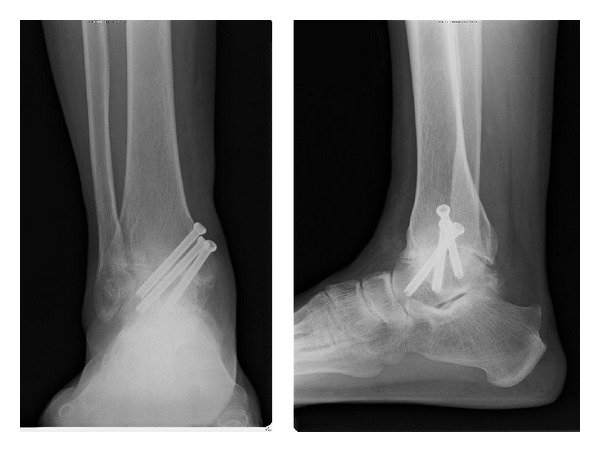
Postoperative radiographs. The bone union is adequate, and no screw loosening is observed.

Although the patient received palliative treatments such as nonsteroidal anti-inflammatory drugs, steroid injection, and rehabilitation, his right ankle pain persisted. Therefore, we then provided an arthroscopic ankle arthrodesis. A medial portal was first made and peeled cartilage of both tibia and talus was observed when we looked into the joint by a scope; otherwise the color of the cartilage was normal (Figure 3(a)). The anterior cartilage was worn and eburnated. Then, a lateral portal was made, and remaining cartilage and anterior osteophytes were removed using a punch or shaver (Figure 3(b)). Dimples were made on the tibial and talar subchondral bone, and bleeding from these dimples was confirmed (Figure 3(c)). The remaining cartilage was removed and more of such dimples were created to completely fuse the tibia and talus (Figure 3(d)). After confirming that all the subchondral bone was removed, the ankle was fixed using three cannulated titanium screws (Stryker Orthopaedics, Mahwah, New Jersey). These screws were inserted from the medial condyle of the distal tibia to the lateral process of the talus, talar neck, and the back of the talus, respectively. The ankle was fixed in the neutral position. After the operation, the ankle was placed in a cast for 4 weeks. Two weeks after the operation, the patient was permitted to bear his weight. At one year after the operation, radiographs revealed adequate bone union (Figure 4), and the patient was asymptomatic for pain at his right ankle.

## 3. Discussion 

KBD is an endemic disease and is very rarely seen outside these areas. However, awareness of this disease is important because of the recent overseas migration of the Chinese. The disease involves lesions of the growth plate and articular cartilage and is often observed in both children and their parents [16], although there is no pathological change around the epiphyseal cartilage and epiphyses plate after the epiphyseal cartilage ceases to develop in adult KBD patients [17]. Adult KBD patients should be differentially diagnosed from primary osteoarthritis patients through their history, age, or residence during childhood and characteristic deformities [17]. In this case, there were no characteristic deformities, but he was young, had no past trauma, lived in the endemic area, and had bilateral ankle osteoarthritis without any other associated inflammatory diseases. KBD often continues to progress and results in osteoarthritis. Radiographs of both ankles revealed osteoarthritis and this is not in conflict with KBD. Ankle is one of the most involved joints of adult KBD and bilateral involvement is the feature of KBD. Therefore, he was diagnosed with KBD and initially received palliative treatment, but his pain persisted. Therefore, we decided to use an arthroscopic ankle arthrodesis as a treatment option.

Many different approaches and techniques for ankle arthrodesis have been reported since Schneider first performed this arthrodesis in 1983 [15]. O'Brien et al. described that arthroscopic ankle arthrodesis yielded comparable fusion rates compared to open ankle arthrodesis, with significantly less morbidity, shorter operative times, shorter tourniquet times, less blood loss, and shorter hospital stays [18]. Arthroscopic arthrodesis is also associated with significant improvement in the ankle osteoarthritis scale compared to that obtained with open arthrodesis at 1 year and 2 years [19]. However, this technique is not adequate for patients with disabling deformities because osseous reference points cannot be identified with the use of an arthroscope [20]. In this case, degeneration of the patient's ankles was seen bilaterally, but few deformities of his ankles were noted. He was then considered a good candidate for arthroscopic arthrodesis. We removed the cartilage and fixed his ankle without any complications. Six months after the operation, adequate bone union was observed, and the patient could walk without pain.

There are few reports on the histology of KBD. Mo described that the affected articular cartilage and growth plate cartilage showed degeneration and necrosis with repair and adaptive changes [21]. We investigated the histology of removed cartilage but only observed minimal fibrous changes. The pathological characteristic of child KBD is absence of penetration of blood vessels in growth plate [22]. However, the pathology of cartilage of KBD is similar to osteoarthritis, such as fissure formation, fibrillation, and degenerative alteration [21]. This is consistent with our findings.

In conclusion, we treated a KBD patient with arthroscopic ankle arthrodesis and did not observe any complications. In KBD cases in which few deformities are observed, arthroscopic arthrodesis is a good treatment option; otherwise, KBD patients often have severe deformities.

## References

[1] Okano T (1919). Survey on an endemic disease in the northern district of Korea (chronic progressive polymorphic multiple arthritis). *Tokyo Iji Shin Shi*.

[2] Wang LH, Fu Y, Shi YX, Wang WG (2011). T-2 toxin induces degenerative articular changes in rodents: link to Kaschin-Beck disease. *Toxicologic Pathology*.

[3] Sokoloff L (1989). The history of Kashin-Beck disease. *New York State Journal of Medicine*.

[4] Yao Y, Pei F, Kang P (2011). Selenium, iodine, and the relation with Kashin-Beck disease. *Nutrition*.

[5] Guo X (2008). Progression and prospect of etiology and pathogenesis of Kashin-Beck disease. *Journal of Xi'an Jiaotong University (Medical Sciences)*.

[6] Schepman K, Engelbert RHH, Visser MM, Yu C, De Vos R (2011). Kashin Beck Disease: more than just osteoarthrosis—a cross-sectional study regarding the influence of body function-structures and activities on level of participation. *International Orthopaedics*.

[7] Guo X, Zhang SHY, Mo DX (1993). The role of low selenium in occurrence of Kashin-Beck disease. *Chinese Journal of Control of Endemic Disease*.

[8] Zhai S Investigation on the relationship between Kashin-Beck disease and drinking water.

[9] Allander E (1994). Kashin-Beck disease. An analysis of research and public health activities based on a bibliography 1849–1992. *Scandinavian Journal of Rheumatology*.

[10] Shi XW, Guo X, Ren FL, Li J, Wu XM (2010). The effect of short tandem repeat loci and low selenium levels on endemic osteoarthritis in China. *Journal of Bone and Joint Surgery—Series A*.

[11] Cao J, Li S, Shi Z (2008). Articular cartilage metabolism in patients with Kashin-Beck Disease: an endemic osteoarthropathy in China. *Osteoarthritis and Cartilage*.

[12] Mathieu F, Begaux F, Lan ZY, Suetens C, Hinsenkamp M (1997). Clinical manifestations of Kashin-Beck disease in Nyemo Valley, Tibet. *International Orthopaedics*.

[13] Xiong G (2001). Diagnostic, clinical and radiological characteristics of Kashin-Beck disease in Shaanxi Province, PR China. *International Orthopaedics*.

[14] Zhao Z-J, Li Q, Yang P-Z (2013). Selenium: a protective factor for Kaschin-Beck disease in qing-tibet plateau. *Biological Trace Element Research*.

[15] Schneider D (1983). Arthroscopic ankle fusion. *Arthroscopic Video Journal*.

[16] Cao CX, Zhang YG, Wu SX, Younas MI, Guo X (2013). Association of clinical features of bone and joint lesions between children and parents with Kashin-Beck disease in Northwest China. *Clinical Rheumatology*.

[17] Li Y, Zhou Z, Shen B (2013). Clinical features of Kashin-Beck disease in adults younger than 50 years of age during a low incidence period: severe elbow and knee lesions. *Clinical Rheumatology*.

[18] O’Brien TS, Hart TS, Shereff MJ, Stone J, Johnson J (1999). Open versus arthroscopic ankle arthrodesis: a comparative study. *Foot and Ankle International*.

[19] Townshend D, Di Silvestro M, Krause F (2013). Arthroscopic versus open ankle arthrodesis: a multicenter comparative case series. *Journal of Bone and Joint Surgery - Series A*.

[20] Ogilvie-Harris DJ, Lieberman I, Fitsialos D (1993). Arthroscopically assisted arthrodesis for osteoarthrotic ankles. *Journal of Bone and Joint Surgery A*.

[21] Mo DX Advances in the pathology of Kashin-Beck disease and its relationship with selenium and other elements.

[22] Pasteels JL, Liu F-D, Hinsenkamp M, Rooze M, Mathieu F (2001). Histology of kashin-beck lesions. *International Orthopaedics*.

